# *Zwakala Ndoda*: a cluster and individually randomized trial aimed at improving testing, linkage, and adherence to treatment for hard-to reach men in KwaZulu-Natal, South Africa

**DOI:** 10.1186/s13063-019-3908-0

**Published:** 2019-12-30

**Authors:** Heidi van Rooyen, Tawanda Makusha, Phillip Joseph, Thulani Ngubane, Michal Kulich, Michael Sweat, Thomas Coates

**Affiliations:** 1 Human Sciences Research Council, Durban, South Africa; 2 MRC/Wits Developmental Pathways for Health Research Unit, Johannesburg, South Africa; 3 Faculty of Mathematics and Physics, Charles University, Prague, Czech Republic; 4 The Medical University of South Carolina, Charleston, USA; 5 University of California Global Health Institute, San Francisco, USA

**Keywords:** Men, HIV testing, Linkages to care, KwaZulu-Natal, South Africa

## Abstract

**Background:**

Men in sub-Saharan Africa are less likely than women to get tested for HIV, less likely to present for treatment, less likely to be maintained in treatment, more likely to have detectable viral load, more likely to transmit HIV with unprotected intercourse, and more likely to progress to AIDS and die sooner from HIV. The ultimate objective of this research is to provide evidence-based strategies to improve HIV testing and treatment of HIV-infected men.

**Methods:**

This study is being conducted in the Greater Edendale Area and Vulindlela region in KwaZulu-Natal, South Africa. It is a two-stage design of a cluster-randomized trial and an individual randomized trial to test how structural and individual-level interventions address the demand-side factors that affect HIV testing and treatment for hard-to reach, high-risk men. It combines male-focused mobilization, community-based mobile HIV testing services, and a small incentive to determine if the strategies singly and in combination can result in more men diagnosed with HIV, and more men linked to and maintained in care with undetectable viral load.

**Discussion:**

A priority for sub-Sahara Africa is developing and evaluating novel and cost-effective strategies for identifying hard-to-reach groups such as men, linking them to HIV testing and care services, and maintaining them in care to the point of viral suppression. We propose a combination prevention intervention that addresses men’s individual, interpersonal, and structural barriers to testing and care. This includes male-led mobilization to encourage uptake of testing and treatment, male-focused testing venues, male-only counselors, developing counseling models that are flexible and responsive to men, and strategies for adhering to clinic visits without missing work and navigating the healthcare system. By thoughtfully combining male-focused mobilization, and testing and addressing some of the barriers to male engagement with health facilities, this study hopes to add to the growing evidence base about how to reach, test, link, and maintain a hard-to-reach group such as men in HIV treatment and care services.

**Trial registration:**

ClinicalTrials.gov, NCT03794245. Registered on 4 January 2019.

## Background

Men in sub-Saharan Africa are less likely than women to get tested for HIV, less likely to present for treatment, less likely to be maintained in treatment and have undetectable viral load (VL), and more likely to progress to AIDS and die sooner from HIV [1–5]. Currently, uptake of testing, linkage, and treatment falls below the levels required to decrease new infections in high-prevalence countries such as South Africa. All available analyses demonstrate that < 17% of HIV-positive individuals in South Africa know that they have HIV, are linked to care, and are maintained in care to reach VL suppression [6]. Doubling antiretroviral therapy (ART) coverage, even to 35%–40%, could reduce HIV infections by up to 40% [7]. The effects are even greater in high-prevalence areas such as KwaZulu-Natal (KZN) [7, 8]. Strategies for engaging the general population, and particularly hard-to-reach groups such as men [2, 4], through all steps of the treatment cascade are critical to epidemic control efforts in South Africa.

Several factors contribute to why men are a hard to reach population. HIV prevention and treatment programs have a blind spot regarding men [1, 2, 9, 10]. More than 30 years into the epidemic, interventions and research focusing on the prevention and care needs of men are notably absent [1, 2, 9–12]. Our attention thus far on women and girls is without dispute. Gender inequality is a key driver that impacts women’s health and access to HIV services and creates specific vulnerabilities for women to HIV infection [13, 14]. However, framing gender as women’s health means we have failed to understand how gender affects and drives the burden of ill health for men [9]. When men are included in HIV prevention and treatment, the focus is frequently on men as the problem (i.e. as transmitters of HIV), with outcomes that focus specifically on improving women’s health, not that of men [15, 16].

Male gender norms discourage men from engaging in testing and treatment [1, 11, 17, 18]. Unhealthy constructions of masculinity and male gender norms associated with toughness and control, sexual prowess, and heteronormativity as a way of asserting manhood can deter men from engaging with HIV services [2, 19–21]. Low testing and treatment engagement is fueled by perceptions of HIV as a threat to notions of masculinity, thus preventing men from testing early enough, disclosing their status, acknowledging their symptoms, or engaging with HIV treatment services [22, 23]. In Malawi, masculine ideals that require men to portray an aura of respectability, financial success, and as providers for their families are barriers to men engaging with testing and treatment services when they should [5, 24].

Key barriers to male engagement in HIV prevention and treatment include social and structural factors. HIV-related stigma and fear of disclosure leads to delays in HIV testing and treatment as well as poor adherence to medication among men [24–28]. Fear of ART side effects as well as misconceptions around the benefits of early diagnosis, care, and treatment can keep people who are living with HIV out of care [12, 29, 30]. Micro-structural factors such as poverty and employment migration keep men away from their partners and families for long periods of time [24]. This absence may make them more vulnerable to HIV infection due to sexual exposure and drug and alcohol use, and may delink them from local health services [3, 31, 32]. Further, health services are not considered male-friendly spaces, with operating hours that often clash with work obligations and provider attitudes that may lack sensitivity to men’s needs, further alienating them [29, 30, 33]. As a result, men have fewer opportunities and disproportionately poorer access to HIV prevention, care, and treatment services.

Existing evidence points to several strategies that could address male involvement in HIV prevention and treatment programs. Community mobilization has successfully changed gender inequitable norms through engaging men to question traditional masculinity and support each other to change social inequalities [34–39]. In our work in Project Accept NIMH/HPTN 043, uptake of HIV testing was improved by changing community norms through enhanced community participation, raising community awareness, and partnership building [40, 41]. Community mobilizing interventions have also demonstrated success in addressing stigma for marginalized populations, thus improving access to sexually transmitted illnesses (STI) care and treatment [42–44]. Programs that address male gender norms and masculinity constructions have a positive impact on men’s and women’s relationships, health, attitudes, and behavior [35, 45–48]. Effective community mobilization that addresses restrictive gender norms could motivate men to engage in testing, linkage to care, and treatment.

Community-based HIV testing services (HTS) optimize the treatment cascade by bringing testing closer to men (and women) thereby increasing demand. HTS, both mobile and home-based, have expanded the geographic coverage and reach of HTS (in both urban and rural locations) and addressed some of the convenience factors—time, costs, distance—typically associated with health facility HTS [49–54]. Our work and others have shown that community mobilization, coupled with mobile HTS targeted at venues frequented by men, can have a significant impact on increasing male engagement in treatment and prevention [11, 34, 49].

Economic incentives (EI) could address structural barriers to HIV testing for men [55]. A review assessing the effects of incentives on HIV/STI testing uptake demonstrated higher rates of uptake in the incentivized group in all seven studies [55]. In rural Malawi, Thornton examined a demand-side EI to randomize cash vouchers redeemable for the return of HIV test results and found that the incentive nearly doubled uptake of HIV testing [56, 57]. A study in Cape Town, South Africa found that greater proportions of the men who received an incentive (a food voucher of approximately US$10) were first-time testers (60.1% vs 42.0%) and had advanced disease (14.9% vs 7.5%) compared with men testing at the non-incentivized clinic services [58]. Finally, incentives in community-based settings [58–60] demonstrated more significant differences in uptake rates compared to incentives offered in clinical settings [61–63]. With demonstrated successes in other areas of health [64–68], including HIV, community-based incentives may encourage more men to take up testing [55, 58].

Once tested positive for HIV, individuals need to be effectively linked to care, treatment, and support services for early initiation of ART and sustained engagement in care. The drop-off that occurs at each step in the treatment cascade results in an estimated 2%–30% of those tested retained in care [69, 70]. There is a gendered slant to the treatment cascade: men are more likely to interrupt treatment [5], be lost to follow-up on ART [12, 16], present for treatment later and often start ART with more advanced HIV disease [5, 16], and experience additional complications than women [1]. Community-based testing and point-of-care (POC) diagnostics for HIV could address some of these challenges. The approach is able to effectively identify HIV-positive persons earlier in the course of their disease course and link people to care [71, 72]. A recent systematic review found that people using POC testing were more likely to both receive a CD4+ T-cell (CD4) result and start ART compared to those relying on laboratory-based methods [73]. In a South African study comparing two linkage-to-care strategies (mobile HTS referral letter versus laboratory CD4 count), the most commonly stated barrier to linkage was accessing public healthcare facilities during working hours and/or not getting time off work (41.4%) [71].

Community-based testing and treatment approaches that actively engage men and their communities, promotes men’s involvement, and is responsive to their needs are essential for developing effective responses to the epidemic in high-prevalence sub-Saharan Africa. This paper describes the protocol for the *Zwakala Ndoda* (an *isi*Zulu term inviting men to participate) study designed to respond to these needs in an area of extremely high HIV prevalence and incidence viz., KwaZulu-Natal, South Africa. Our study innovatively combines an evidence-based testing strategy (male-focused mobilization, community-based mobile HTS, and a small incentive) with proven, effective linkage-to-care strategies in one model. Our rigorous design allows us to test the individual and combined effects of novel strategies for HIV testing, linkage, and maintenance in care.

## Methods

### Study setting

This study is being conducted in the Greater Edendale Area (GEA) and Vulindlela region in KZN, South Africa. The area has a combined population of nearly 400,000 people spanning 20 municipal wards characterized by low population density, few infrastructure and developmental resources, high HIV prevalence (31%), high unemployment, and low per capita income (under US$2 per day) [25]. The population is primarily black African (99.3%). Nine of the wards fall under the tribal authority of a traditional leader or chief. Health services are provided through seven health facilities: Edendale Hospital, the main referral facility; two primary health clinics; and three satellite primary health clinics.

### Study aims

The study combines the best of three strategies (male-focused mobilization, community-based mobile HTS, and a small incentive) to determine if the strategies singly and in combination can result in more men diagnosed with HIV and more men linked to and maintained in care with undetectable VL. The specific aims are listed below.

- Aim 1: To test the hypothesis, in a cluster-randomized design, that men-centered mobilization and testing strategies plus a small incentive will result in a substantially higher proportion of men getting tested for HIV (difference at least 30%).
- Aim 2: To test the hypothesis, in an individual randomized design, that POC CD4 testing combined with personalized linkage to care (PLC) will result in a higher proportion of HIV-positive men linked to and maintained in care with undetectable VL than POC alone and that both will be superior to standard of care.
- Aim 3: To integrate the outcomes of the structural and individual-level interventions and evaluate the joint effect of the structural and individual-level interventions on the percentage of HIV-positive men who are effectively treated (tested, linked to care, and maintained with undetectable VL).

#### Research design

**Fig. 1:**
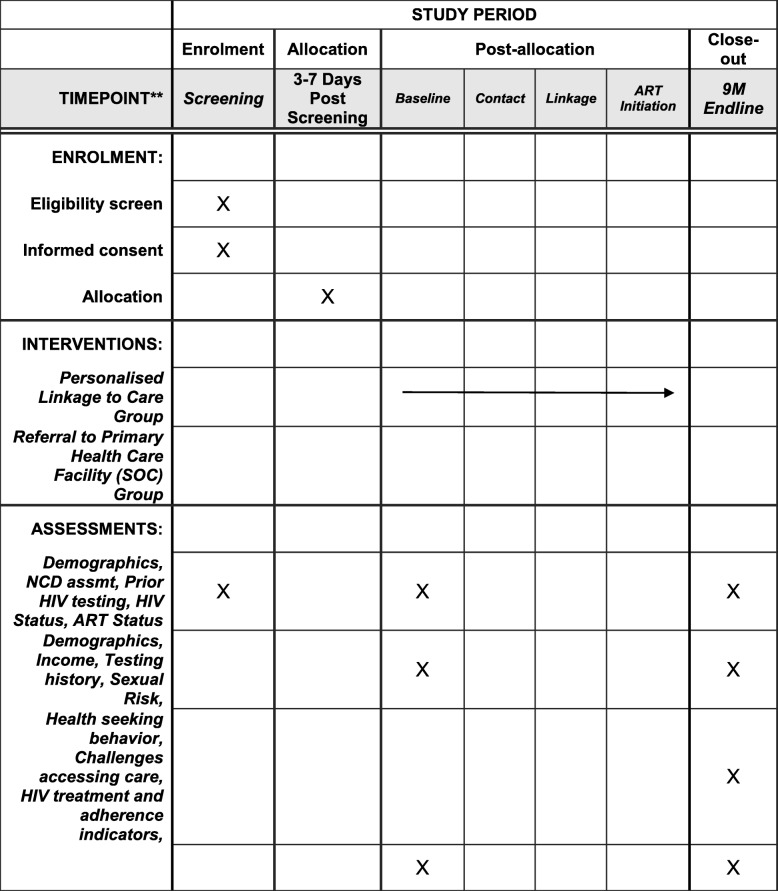
Zwakala Ndoda Intervention SPIRIT *schematic*

This is a two-stage design of a cluster-randomized trial and an individual randomized trial to test how structural and individual-level interventions address the demand-side factors that affect HIV testing and treatment for hard-to-reach, high-risk men. A Standard Protocol Items: Recommendations for Interventional Trials (SPIRIT) diagram is presented in Fig. 1 and Additional file 1.

#### Cluster-randomized study

Eight communities of approximately 5000 persons (four intervention and four control communities) from the GEA and Vulindlela regions will be selected with an equal mix of rural and peri-urban communities to ensure greater generalizability of the intervention in different settings. Each of the communities will be randomized to either men-centered mobilization and testing strategies plus a small incentive or the control condition. Randomization will be performed by the statistical center in a restricted way, so that four communities are assigned to each arm. Randomization code will be written in R and will use the Mersenne-Twister random number generator with seed derived from current system time.

#### Individual randomized study

A sample of 440 HIV-positive men identified though mobile HTS at baseline will be randomized to either standard-of-care referral to ART or POC CD4 testing combined with personalized linkage to care. Randomization sequence will be generated in advance by the statistical center, using the Mersenne-Twister random number generator with seed derived from current system time. Randomization assignments will be placed into sealed tamper-proof numbered envelopes according to the generated sequence and released to study counselors in small batches. The envelopes will be used in the assigned order to reveal randomization results for individual participants. Lost or misplaced envelopes will be registered and tracked. The randomization sequence will be fully non-predictable.

#### Blinding

Due to the nature of the interventions, neither community-randomized nor individual-randomized intervention assignments are blinded to everyone. However, the laboratory results are completely blinded. The study statistician cannot be blinded because the intervention assignments were created by him and is also part of a team that conducts an unblinded review of process outcomes such as enrolment and dropouts in the study. Nevertheless the data processing and analysis will be done in the same impartial way in both intervention groups.

### Research ethics approval and data safety monitoring

The Institutional Review Board of the University of California, Los Angeles (UCLA, 00003962) and the Research Ethics Committee of the Human Sciences Research Council in South Africa (HSRC, REC 3/18/02/15) have approved the study and oversee adherence to the study protocol over time. A six-member Data Safety and Monitoring Board (DSMB), consisting of local and international experts, will monitor the implementation of the trial. National, provincial, district, and municipal health authorities approved the study and its protocol and set the conditions of the standard care practice.

The DSMB will review the effectiveness and scientific validity of the study. The DSMB will provide their evaluation feedback to the principal investigators and study sponsor assessing interim analyses on enrolment progress, protocol fidelity, and participant safety. Concurrently, enrolment progress is reported each trimester directly to the study sponsor. Ongoing harms and adverse monitoring is overseen by the local institutional research ethics committee (REC). A Community Advisory Board (CAB) consisting of community stakeholders, representatives, and leaders has been constituted for this study. Quarterly meetings are held with a CAB consisting of 25 local stakeholders who serve as liaison between research staff and the community, advising on study policies and keeping the community informed of progress. CAB members and study personnel have been trained to identify and report incidents of social harm and adverse events that may occur in the community to the study project manager. Regular feedback meetings are conducted with health authorities. The study project manager is responsible for logging, assessing, and actioning any appropriate remedial steps (onward referral, staff re-training, community messaging, etc.), reporting to the local REC and maintaining a register of incidents, if any, for the DSMB. The study will engage with participants’ preferred clinics for a formal feedback and update meeting on post-trial care. The study team will engage with the department of health and local health facilities to create a database of options (community-based re-supply, active support groups, and accredited health system partners). The study team will share this information with participants and facilitate access to programs for which the participants may be eligible, with referral to active support groups and adherence clubs.

### Research activities and procedures

#### Male-focused community outreach and mobilization (Component 1)

The community outreach team engage traditional leaders and key governmental and local stakeholders to obtain permission to conduct the study in the communities. Information is provided about the study aims and objectives as well as the rationale for including men in the intervention. The team identifies formal (e.g. community halls and churches) and informal social venues (e.g. transport hubs and sporting events) in each of the study communities where men are mobilized to participate in the study. At both venues, four community-based mobilizers (CBMs) conduct activities such as dialogues, social games, and distribution of pamphlets and edutainment to encourage men to test and link to treatment (see Table 1). Through these social networks, early adopters are identified and encouraged to test. Some of the early adopters are invited to be community champions (CC). CCs work with study staff to identify other men in their networks who are invited to participate in the intervention. These formal spaces are used for mobilization activities but also as possible spaces where mobile HIV testing is conducted. In addition, men identified through these venues are given information about the mobile HTS schedule and venues.

**Table 1:** Men-centered mobilization strategies

Social games and edutainment	Different games are organized monthly per intervention community. These games include street soccer, field soccer, fun run/walk, card games, etc. These also include screening of soccer games on a big screen in taverns if a community Wi-Fi hot spot is available. While men participate in these games, health messages are distributed and testing is provided through the mobile van.
Men’s dialogues	The team organizes dialogues as a safe space for men to discuss matters pertaining to their health. Men are encouraged to be open about issues concerning them. Video clips of topics such as “AIDS in our community, Living positive with HIV/AIDS, Phuza Wise, Help stop women abuse, Alcohol and you” developed by a local communications non-governmental organization called Soul City (*https://www.soulcity.org.za*) are played to prompt discussion about men and their health. HTS staff are available to provide testing before and after each dialogue.
Pamphlets	Large amounts of pamphlets with study information are printed and distributed in taxi rank, taverns and sheebens, busy intersections, places of worships, and many other social venues. Posters are also pasted at strategic venues where are men are frequenting. Some of these posters provide general HIV testing FAQs and others are provide testing van schedule.

*HTS* HIV testing services

In addition to recruiting men to testing through formal and informal venues, CBMs go from house to house in the study communities to talk to men about healthy living and the importance of screening for HIV and other diseases. These CBMs also visit busy road intersections and talk with people who are passing by and distribute study information using pamphlets and appointment cards. In both these instances, men are provided information regarding when testing is likely to happen in their communities.

#### Male-focused and delivered mobile HTS (Component 2)

The mobile HIV-testing services teams coordinate with CBM teams to ensure that testing happens according to the testing schedule. Copies of the testing schedule are distributed to community working groups, community-based outreach volunteers, local police stations, local health centers, and other community centers deemed appropriate by the study team.

Two HTS teams (four male nurse counselors and one driver per team) deliver easy-to-access mobile HIV testing through two mobile caravans. A mobile HTS team circulates through a range of male-centered locations in the community on a regular schedule of days and times suitable for men. Two teams serve the four intervention communities, with each team being responsible for two intervention communities over the course of the intervention. The team is supervised by a Counselor Supervisor.

CBMs are stationed in the vicinity of the men-centered spaces and engage men individually and in groups about the study. Mobilizers also inform men that a small incentive of R50 cell phone airtime (approximately US$5) would be received at completion of testing, regardless of HIV status. Other men in the vicinity of the mobile facility, but not necessarily frequenting the venues, are also encouraged to test by study staff. Recruited men are handed over to the study counselor at the mobile caravan for a fuller explanation of the study, informed consent, and completion of the HTS process.

Participants give consent for on-site POC procedures for screening for HIV and non-communicable diseases (NCDs) using rapid and POC tests. No specimens are retained for HIV and NCDs testing. In the event of a discordant HIV test result, a Dry Blood Spot (DBS) specimen will be collected from the participant for a laboratory confirmatory test to confirm serostatus. No specimens are retained for confirmatory tests. Participants who are eligible for enrolment (HIV-positive and not on antiretroviral treatment) and who consent to enrolment provide a DBS for baseline VL measures. Consent is provided for participants to be re-contacted at three and six months for study contact visits and for an exit visit at nine months after enrolment where participant exit survey data and end-line VL will be collected. Participants consent for their data to be stored and for secondary use of their data if such use is approved by the REC.

After consent is obtained, the study counselor completes the utilization form on the mobile phone. The form includes basic sociodemographic information and information about previous testing behavior as well as engagement in HIV treatment and prevention and related health activities. If the male is not willing to participate, he will still complete the utilization form to allow for comparison of participants with non-participants.

#### Brief risk-reduction counseling

In our Uganda study, we showed that briefer modes of HIV risk-reduction counseling are likely to be equally effective as extended counseling and testing [74]. We adapted this briefer risk-reduction counseling method (10–23 min) by incorporating appropriate messages into the counseling that would addresses men’s masculinity concerns and gender beliefs about testing and treatment (see Table 2 for a breakdown of each session). The approach is male-friendly: services are provided to men in their social spaces and at their convenience; sessions are shorter and tailored to address their particular concerns; men do not have to wait in long queues; and all screening services are offered by male counselors.

**Table 2:** Brief risk reduction counseling model

	Length of time (min)
*Protocol Component 1: pre-test counseling*	
1. Introduction of HIV testing to patients	1–5
First session time	Total 1–5
*Protocol Components 2–4: disclosure of HIV-negative results*	
2. Provide HIV-negative test result	1–2
3. Develop risk reduction plan	1–2
4. Discuss disclosure, discordance, and partner testing	1–2
Second session time	Total 3–6
*Protocol Components 5–7: disclosure of HIV-positive results*	
5. Provide HIV-positive test result and identify care resources	2–4
6. Discuss disclosure, discordance, and partner testing	2–4
7. Develop risk reduction plan	2–4
Second session time	Total 6–12

During the pre-test counseling session, the counselor explains the clinical and prevention benefits of testing, the right to refuse, the follow-up services that will be offered, and, in the event of a positive test result, the importance of disclosure to others who may have been exposed to HIV infection. If the patient has never tested for HIV, the counselor explains why it is important to know one’s HIV status and then offers the test.

Post-test counseling is tailored to the outcome of the test (positive/negative) and retain key messages (e.g. disclosure and partner testing, risk reduction, linkage to care, availability of care). The HIV-positive patient counseling session focuses on emotional support, HIV risk reduction, disclosure of HIV status, follow-up care available and referral, and partner notification and testing. Counseling for the HIV-negative patient focuses on HIV risk reduction, disclosure of HIV status, and partner notification and testing.

#### HIV testing

HIV testing is conducted using blood collected by finger-stick with a sterile lancet and using a serial testing algorithm for rapid testing according to 2015 South African National HIV Test Services guidelines [75]. All specimens are first tested with one assay (Test 1 or screening test) and specimens that are non-reactive are considered HIV-negative and reported as such. Any specimens that are reactive on the first assay (Test 1) are tested again using a different assay (Test 2 or confirmatory test). For specimens that are reactive on both the first and the second assays, results are reported as HIV-positive. Specimens that are reactive on the first assay but non-reactive on the second assay will receive an ELISA laboratory test and be recorded as discordant. In the case of discordant results, the individual is given the schedule of mobile HTS testing locations and asked to return in seven days [7] for their HIV results.

Participants are triaged according to their HIV test result. HIV-negative participants will have completed their visit, as they are not eligible for further study participation and, if required, referrals to relevant services are made. Study counselors will explain to HIV-positive men in the intervention communities that they are eligible for the randomization and the next phase of the study, if interested. All HIV-positive participants are asked for locator information (addresses and alternate/additional contact phone numbers) so if randomized into the PLC arm they can be contacted. Permission is obtained to call and/or text the participant to arrange and keep in contact for PLC.

#### Screening for non-communicable diseases

In order to destigmatize HIV testing for men, a variety of screening services for NCDs are provided alongside the screening for HIV. Procedures for hypertension, diabetes, obesity, and STI are explained to interested men, the relevant tests conducted, and results provided. Appropriate information on these conditions as well as follow-up referrals are made to nearby health facilities if further care is indicated.

#### Control communities

Control communities receive the current standard of care HTS services. All primary healthcare facilities in South Africa provide client-initiated counseling and testing (CICT) and provider-initiated counseling and testing (PICT). CICT involves individuals or couples/sexual partners actively seeking HIV testing and counseling at a facility that offers these services, while PICT is initiated and recommended by healthcare providers to all clients attending healthcare facilities as a standard component of medical care. All counseling includes the provision of pre-test information sessions conducted with groups, couples, or individuals, the HIV test itself, and post-test counseling. The standard of care also involves the promotion of HTS through flyers in clinics and the service is offered free of charge during office hours, predominantly on weekdays.

#### Quality assurance of HTS

Rapid, on-site HIV testing has the potential to be a useful and advantageous testing strategy in community-based or outreach settings. The intervention performs HIV testing according to the South African national rapid testing algorithms with ongoing external quality assessment (EQA). All staff are fully trained in performing the rapid testing algorithm. CD4 counts are done by POC tests with ongoing EQA. VL testing are done at a laboratory with EQA participation. Training in rapid testing technology was done as part of the 10-day counseling course for counselors.

In addition to covering all of the didactic portions of the counseling and testing process, counselors practice using the test kits themselves with known HIV-positive and HIV-negative sera to ensure that they are performing and reading the tests correctly. Counselors also practiced with mock patients under the supervision of the Counselor Supervisor. Supervisors periodically observe counselors in the HTS process, using a standardized checklist, to ensure that all parts of the procedure are being followed. Counselors are recertified bi-annually with panels of HIV-positive and HIV-negative sera. They are expected to achieve 100% correct results; if they fail to do so, the counselor is taken out of the study, retrained and recertified, and monitored closely to ensure correct adherence to testing protocols. We also conduct daily test kit validation to ensure quality test results. For each mobile unit, we conduct a known positive and known negative validation. We expect perfect concordance of results. If we do not achieve this, the batch is identified and set aside. A new batch of test kits will be validated and used for further testing.

#### Personalized linkage to care (Component 3)

In this third component of the intervention, HIV-positive men are randomized into two arms: (1) standard of care HIV follow-up; or (2) POC CD4 testing with male-centered PLC.

#### POC CD4 testing with male-centered PLC

HIV-positive participants randomized into the PLC phase of the study receive POC CD4 testing. The study nurse conducts same-day CD4 testing by taking a small blood sample obtained from the participant’s fingertip by a lancet. The date of the HIV and CD4 test and symptoms reported by the participant are provided in a one-page letter to the clinic to facilitate ART initiation. At the end of the counseling session, the nurse counselor schedules a follow-up visit for the participant and their study assigned PLC clinic-based champion (CBC). The CBC coordinates schedules with the participant and visits the HIV clinic with the participant within two weeks of enrolment. PLC services are tailored for individual clients and include pro-active case-management counseling to discuss and reduce barriers to linkage to care, adherence, and the importance of disclosure. It also comprises CBC accompaniment of the participant to help them navigate the health systems and extended hours access to the ART clinic.

#### Accompaniment to the HIV clinic by the clinic-based champion

The PLC CBC discusses with the participant the usual means of travel to the clinic and encourages the participant to attend the clinic. As appropriate, the CBC may travel with the participant to the clinic by public transport or meet the participant at the clinic. At the clinic accompaniment visit, CBCs answer questions and provide support for HIV care. If several accompaniment visits are required before linking to care, the CBC may make these visits with the participant.

#### Extended clinic hours for men (happy hour)

The PLC team leader negotiates with health services to ensure that extended hours of operation for obtaining ART and services are made available to PLC participants who have conflicting engagements during regular HIV clinic hours. This service (called happy hour) is already provided to young people by the Department of Health. A sample of clinics are opened once a week during 17:00 to 20:00, especially to cater for men. This male-only HIV service allows the PLC participants to feel more comfortable in accessing services and discussing their questions and concerns more freely with the staff at the after-hours HIV clinic. The after-hours HIV clinic will offer all services normally available at a standard HIC clinic during regular work hours including HIV testing, counseling, and ART medicines.

#### Regular follow-ups by the clinic-based champion

Further follow-up visits may be scheduled for three and six months after the initial visit, if the participant has not established care at an HIV clinic. At the follow-up visits, CBCs answer questions about barriers to care and difficulties with adherence as well as provide support for HIV care. Participants complete a questionnaire on their experience accessing care, including barriers to care, risk behavior, HIV clinical care, and knowledge of HIV treatment and prevention. Examination of HIV care documentation (e.g. registration card from HIV care clinic) and recording of medications (e.g. HIV care medications and ART) takes place.

CBCs also link men to appropriate referrals and support, including arranging opportunities for men to come together for support groups with other men to talk about and discuss issues of disclosure, adherence, and retention in care.

#### HIV standard of care

HIV-positive men randomized into the standard of care arm are referred to the study counselor. All participants who get assigned to standard of care receive the HIV and NCDs testing results slip that is used as a referral letter with all testing outcomes written on it. The lay counselor provides information about HIV treatment and encourages the participant to visit the nearest clinic or any healthcare service of their choice for further clinical attention. Participants get information about being contacted by telephone at three and six months in preparation for the nine-month follow-up visit. The telephone contact at three and six months is for tracking purposes to maintain contact with the participant. At nine months, the participant is invited to participate in another short survey and DBS specimen collection. Another consent process with the participant is solicited at this point.

### Study outcomes

Aim 1: Outcome measures compare men-centered mobilization and testing strategies in intervention communities to those in control communities.

#### Primary outcomes

1. Proportion of men getting tested for HIV in the last 12 months. This is to find out how many men have been tested for HIV in the last 12 months.

#### Measurement of Aim 1 outcomes

Conduct post-intervention assessments in intervention and control communities (n = 1600, 8 communities × 200).

- Post-intervention random survey data (focusing on self-reported testing history, other wellness-related data, health-seeking indicators, etc.); and specimen samples from men living in the study communities. We will be able to look at reported HIV status versus actual status.

Aim 2: Outcome measures compare enrolled participants assigned to PLC to those in standard care during the intervention.

#### Primary outcomes

1. Proportion of men linked to care for first clinic visit in the last nine months. This is to establish how many enrolled men accessed healthcare during the intervention.
2. Proportion who remained in care in the last nine months. This is to establish how many enrolled men completed scheduled clinic visits during the intervention.
3. Proportion with undetectable VL at exit. This establishes how many enrolled men have suppressed VL at exit.

#### Measurement of Aim 2 outcomes

This uses information collected at the nine-month exit interview.

- We will conduct facility level chart abstraction to confirm linkage to care for those enrolled in the study and laboratory DBS VL assessment to establish viral suppression.

### Theoretical rationale

Our combination prevention intervention is informed by the social-ecological framework which demonstrates how effective intervention can effectively address individual, socio-cultural, and program or health facility factors that influence individual health decisions. At the individual level, men’s testing and treatment decisions require careful balancing of perceived benefits against constraints of adopting these health behaviors. Men report testing decisions as comprising a struggle between fears of the consequences of not testing (illness and death) and fears of the consequences of HIV testing (stigma and loss of status as a man). For men, there may also be fears that testing exposes their hidden sexual activities [1, 2]. Socio-cultural factors affect men’s decisions regarding healthcare [16]. Prevalent gender norms are important determinants of both decisions to undergo HTC for men and of subsequent progression through the HIV care pathway.

Social networks create powerful conduits for both support and discouragement for men on this pathway. The influences of other men in the social network regarding testing and treatment and how this feeds into ideas of masculinity as well as the influence of sexual partners, family members, and friends also factor into men’s decisions regarding testing and treatment. Health facility factors also shape the individual’s cost-benefit analysis of seeking and sustaining testing and treatment. The distance to medical services, financial and opportunity costs of travel, and the quality of care, such as waiting time and the attitude of providers, are all important factors in testing uptake and treatment compliance for men. Men thus formulate personal options and behavioral intentions while negotiating the social environment and the parameters of testing and treatment programs and they do so against a broader structural environment that in much of sub-Saharan Africa includes entrenched poverty, food security, and weakened health systems [76]. Our multi-level intervention attempts to address all three levels that affect men’s decision-making regarding testing and engagement in care.

### Data management and adverse event reporting

The assessments in the study are administered using electronic mobile data capture. The mobile data collection platform allows for offline data collection. Collected data are uploaded to the server once the network connection is re-established. Checks are placed to ensure correct information has been entered. Data from laboratory testing (i.e. plasma HIV VL) is collected by participant ID number and merged into the database. Information from the questionnaires collected via the mobile phone is uploaded to a secured server. The de facto standard for securing network traffic is Secure Sockets Layering (SSL). This technology is fully supported by the handsets used in this study and ensures that all data transferred between the device and the server is encrypted.

Similarly, when reviewing, exporting, or managing data, all communications between the browser and server are encrypted. All data are encrypted. Servers are secured by firewalls to prevent unauthorized access and denial of service attacks. Data are protected from virus threats using antivirus technology. The study database is backed up regularly. All personal identifying data will be destroyed five years after the study is completed, in compliance with the regulatory requirements of the study funder. The team is committed to ensuring that identifying data, including GPS, are secure until the time of destruction.

#### Quality assurance

To assure data accuracy and completeness, the mobile data collection platform is programmed to require data submission on all data fields. Skip patterns and logic branches are programmed to ensure that valid data are collected. Range checks are programmed on key fields to ensure data consistency. Operational data are collected for cross-check, data tracing, and validation purposes. To assure data security, all hard-copy data are stored in locked cabinets in a locked office and all electronic data are password-protected and stored on a secure server in a secure facility. To ensure protocol compliance, the Project Director conducts periodic observations of study procedures (e.g. observing the informed consent process, a face-to-face interview, or HIV test counseling), conducts periodic “retraining” on key study procedures using role-plays during staff meetings, and produces a regular quality assurance report for the PI.

### Data analysis

#### Aim 1: Cluster-Randomized component

We will test the hypothesis that the community-wide proportion of men who were tested for HIV in the past year is at least by 10 percentage points (on an absolute scale) larger in communities randomized to the active intervention compared to the standard-of-care arm. The analysis will be based on testing reports provided by men included in the post-intervention assessment. Empirical proportions of tested participants will be calculated for each community and compared between the study arms by a two-sample Welch t-test. We will test the hypothesis that the difference in proportions is ≤ 0.1 against the alternative that the difference is > 0.1 at the one-sided level of 0.05.

#### Power calculations for Aim 1

We assumed 30% annual testing rates in the standard arm (similar to testing rates observed in Project ACCEPT in the community-based HTS arm in Vulindlela site) and between-community variance in testing rates 0.007 (estimated from Project ACCEPT’s Vulindlela data). Then, the sample size of four communities per arm, with 200 men sampled from each community, provides the following power for detecting intervention effects: power 0.05 for 10% difference (30% vs 40%, null hypothesis); power 0.36 for 20% difference (30% vs 50%); power 0.86 for 30% difference (30% vs 60%); and power 0.95 for 35% difference (30% vs 65%). Thus the study is powered to detect differences in testing rates that are substantially > 20% and therefore likely to be of practical significance. The power for detecting differences of ≥ 30% is large enough to be reasonably certain that such effects are not missed.

#### Aim 2: Individually Randomized component

We will test the hypothesis that the proportion of HIV-positive men who were linked to care and maintained with undetectable VL nine months after enrolment will be the same in the POC + PLC arm as in the standard-of-care arm. The analysis will be based on men who were randomized and who completed the follow-up visit. We will use asymptotic tests for log odds ratios performed at the two-sided level of 0.05. Confidence intervals will be obtained by the same method.

#### Power calculations for Aim 2

Assuming that the rates of linkage to care and undetectable VL are 33% in the standard arm and 50% in the POC + PLC arm (odds ratio 2.0), the sample size of 175 individuals per arm yields 88% power to detect the effect of POC + PLC over standard of care. Adjusting for 20% attrition yields the sample size of 220 per arm.

#### Secondary analyses for Aim 2

We will carefully investigate the effect on attrition on the primary analysis of Aim 2. We will compare the men who do and do not present for the final visit to assess potential bias in the analysis of the primary endpoint of Aim 2. We will evaluate the attrition effect by multiple imputation of missing data using baseline characteristics of the individuals. We will compare HIV-positive men identified in Aim 1 who do and do not enroll in the Aim 2 study.

#### Aim 3: Integrating the outcomes of the structural and individual-level interventions

We will estimate the joint effect of the structural and individual-level interventions on the percentage of HIV-positive men who are effectively treated (tested, linked to care, and maintained with undetectable VL) by estimating the percentage of HIV-positive men who have been tested in male-centered mobile HTS versus control arms using data on HIV testing and HIV status of the post-intervention assessment participants from Aim 1, and estimating success rates of undetectable VL in SOC and POC + PLC arms in Aim 2 (adjusted for attrition if needed). These estimates will be multiplied to estimate overall success rates of undetectable VL among HIV-positive men in all four combinations of the two interventions. Confidence intervals will be also calculated. We will assume that the interventions do not interact with each other since each targets a different stage in the process leading to successful HIV treatment of HIV-positive men. Given population size and HIV prevalence, we will be able to estimate the number of men who would be identified, treated, and maintained on treatment under each of the possible intervention combinations. A sensitivity analysis will be performed to evaluate the impact of potential non-independence of intervention effects.

A full factorial design is not feasible because one of the interventions is applied at the community level and the other at the individual level; success at the first of the interventions (being tested) is a condition of enrolment into the other intervention. This brings bias into the randomization of the community-level intervention. This design could only be conducted by randomizing whole communities to the four combinations of the interventions, but that would be too costly.

#### Cost-effectiveness analysis

We will examine effectiveness in terms of: (1) behavioral changes resulting from HIV testing and counseling (which reduces HIV transmission and acquisition); (2) increases in life-expectancy and quality of life from engagement and retention in care; and (3) reductions in HIV transmission, and thus morbidity and mortality from downstream infections, derived from reduced HIV infectivity of those on ART. We will contrast standard of care versus exposure to (1) male-centered mobilization + mobile HTC and (2) combined POC CD4 + personalized linkage to care. For each, we will use Bernoulli-Process models for HIV transmission, extension of life, and enhancement of quality of life.

#### Sensitivity analysis

We use Monte Carlo and Latin Hypercube simulations with @Risk™ software for sensitivity analyses. Confidence intervals and point estimates from study data will be fitted to distribution functions. Convergence will occur when the addition of model iterations changes the average and standard deviation of the output by < 1.5%. Correlations between model parameters will be identified to better fit the model.

## Discussion

The ultimate objective of this research is to provide evidence-based strategies to improve treatment of HIV-infected men. A priority for sub-Sahara Africa is developing and evaluating novel and cost-effective strategies for identifying hard-to-reach groups such as men, linking them to HIV testing and care services, and maintaining them in care to the point of viral suppression [77]. In this study, we are combining the best of these strategies (male-centered community outreach and mobilization followed by male-centered and delivered mobile HIV-testing services plus a small incentive, and finally personalized linkage to care) to determine whether the strategies singly and in combination can result in more men diagnosed with HIV as well as more men linked to and maintained in care with undetectable VL.

A growing number of interventions are beginning to target men and boys in their prevention work and rigorously evaluating the impact, with mostly positive results [2, 16]. This study combines structural (community) and individual-level interventions and integrates the two to address our objective of maintenance in care to the point of viral suppression, adding to the few rigorous evaluations of male-focused interventions in South Africa.

Community-based testing and treatment approaches that actively engage men and their communities, promote men’s involvement, and are responsive to their needs are essential for developing effective responses to the epidemic in high-prevalence sub-Saharan Africa. Research increasingly shows that men prefer community-based events where they would have the opportunity to ask questions and where they can talk about the reality of their lives [48]. The low rates of treatment engagement in South Africa and KZN, in particular, could limit the potential impact for these novel treatment-based strategies to reach the communities where they are most needed [78].

A growing number of studies point to two reasons for men’s low involvement in HIV services. The first relates to the gender inequalities and associated gender norms about masculinities that encourage men to act in ways that put themselves and their sexual partners at risk of contracting HIV [5, 16]. In South Africa, and other parts of sub-Saharan Africa, these include a range of male sexual behaviors and practices, such as an unwillingness to use condoms or get tested for HIV, as well as engagement in multiple and concurrent partnerships increase men’s likelihood of contracting HIV and transmitting it to their female partners [3, 13, 79].

Addressing harmful gender norms that inform these practices by reaching out strategically to men may be one of the best ways to protect women (and men themselves) [3, 4]. By placing men at the center of this study, we do not seek to exclude women but acknowledge that men and women may need different approaches to motivate HIV testing and linkage to and maintenance in care. Further, engaging men in testing and treatment could keep them healthy for longer periods, thus having a positive impact on the economic circumstances of their families and potentially alleviating the care burden on women [9, 11].

The second factor for low male involvement in HIV prevention and care points to the need for improved health system policies [5], programs, and service delivery strategies to ensure better provision of HIV services to men [16]. Finally, when men are included in policies and programs, they are all too often seen only as the problem and as vectors of HIV [2]. As a result, limited attention is placed on how men can be meaningful and supportive partners and how to effectively link men into the HIV prevention and care cascade. This study addresses this important gap by developing targeted strategies that engage men both as agents of change and as holders of the right to health, including especially HIV and AIDS services.

We propose a combination of prevention intervention that addresses individual, interpersonal, and structural barriers to male involvement in testing and care and is responsive to their needs [29, 30, 33]. Our innovative male-centered combination prevention interventions include: (1) male-led mobilization to encourage uptake of testing and treatment; (2) male-focused testing venues convenient and accessible to men and their work obligations; (3) male-only counselors who will counsel other men; (4) counseling and mobilization messaging that address gender norms and masculinity ideals – promoting the benefits of good health for earning income and supporting one’s family, the importance of being a strong role model for children, especially sons; (5) developing counseling models that are flexible and responsive to men and their work demands; (6) ways to get support from fellow men; and (7) strategies for keeping clinic visits without missing work and navigating the healthcare system. By thoughtfully combining male-focused mobilization and testing and addressing some of the barriers to male engagement with health facilities, this study hopes to add to the growing evidence base about how to reach, test, link, and maintain a hard-to-reach group such as men in HIV-treatment and care services.

## Trial status

Screening and recruitment for the trial began on 30 August 2017. As of 30 November 2018, the study team has been in the field for 15 months with 4473 participants screened (4179 screened for NCDs and tested for HIV, 88 tested for HIV only, and 206 screened for NCDs only). A total of 249 participants have been enrolled for the personalized linkage to care intervention trial within the study. It is expected that enrolment will continue until 30 April 2020.

## Supplementary information

**Additional file 1.** SPIRIT 2013 Checklist: Recommended items to address in a clinical trial protocol and related documents.

## Data Availability

The datasets generated and/or analyzed during the current study are available from the corresponding author on reasonable request.

## Abbreviations

ART: Antiretroviral therapy
CAB: Community Advisory Board
CBC: Clinic-based champion
CBMs: Community based mobilizers
CC: Community champions
CD4: CD4+ T-cell
CICT: Client-initiated counseling and testing
DBS: Dry Blood Spot
DSMB: Data Safety and Monitoring Board
EI: Economic incentives
EQA: External quality assessment
GEA: Greater Edendale Area
HTS: HIV testing services
KZN: KwaZulu-Natal
NCDs: Non-communicable diseases
PICT: Provider-initiated counseling and testing
PLC: Personalized linkage to care
POC: Point-of-care
SPIRIT: Standard Protocol Items: Recommendations for Interventional Trials
SSL: Secure Sockets Layering
STI: Sexually transmitted illnesses
VL: Viral load
